# Dr. Lewis Nelson

**Published:** 1912-05

**Authors:** 


Dr. Lewis Nelson, an instructor in the Harvard Medical School died April 15, 1912, from an infection with the bacillus aerogenes capsulatus, received in some researches that he was making.

